# A Genetic Network Underlying Rhizome Development in *Oryza longistaminata*

**DOI:** 10.3389/fpls.2022.866165

**Published:** 2022-04-05

**Authors:** Wenfei Li, Shilai Zhang, Guangfu Huang, Liyu Huang, Jing Zhang, Zheng Li, Fengyi Hu

**Affiliations:** State Key Laboratory for Conservation and Utilization of Bio-Resources in Yunnan, Research Center for Perennial Rice Engineering and Technology of Yunnan, School of Ecology and Environmental Science, School of Agriculture, Yunnan University, Kunming, China

**Keywords:** *Oryza longistaminata*, rhizomes, development, genetic networks, quantitative trait loci

## Abstract

The rhizome is an important organ through which many perennial plants are able to propagate vegetatively. Its ecological role has been thoroughly studied on many grass species while the underlying genetic basis is mainly investigated using a rhizomatous wild rice species—*Oryza longistaminata*. Previous studies have revealed that the rhizome trait in *O. longistaminata* is jointly controlled by multiple loci, yet how these loci interact with each other remains elusive. Here, an F_2_ population derived from *Oryza sativa* (RD23) and *O. longistaminata* was used to map loci that affect rhizome-related traits. We identified 13 major-effect loci that may jointly control rhizomatousness in *O. longistaminata* and a total of 51 quantitative trait loci (QTLs) were identified to affect rhizome abundance. Notably, some of these loci were found to have effects on more than one rhizome-related trait. For each trait, a genetic network was constructed according to the genetic expectations of the identified loci. Furthermore, to gain an overview of the genetic regulation on rhizome development, a comprehensive network integrating all these individual networks was assembled. This network consists of three subnetworks that control different aspects of rhizome expression. Judging from the nodes’ role in the network and their corresponding traits, we speculated that *qRHZ-3-1*, *qRHZ-4*, *qRHI-2*, and *qRHI-5* are the key loci for rhizome development. Functional verification using rhizome-free recombinant inbred lines (RILs) suggested that *qRHI-2* and *qRHI-5*, two multi-trait controlling loci that appeared to be critical in our network analyses, are likely both needed for rhizome formation. Our results provide more insights into the genetic basis of rhizome development and may facilitate identification of key rhizome-related genes.

## Introduction

Rhizomes are modified subterranean stems that grow horizontally and can produce roots and shoots on their nodes, enabling vigorous asexual proliferation in various perennial species (Gizmawy et al., 1985; Li et al., 2022). From a physiological point of view, the rhizome is also the main energy storage organ of many perennial plants and plays a determinate role in their survival in harsh environment (Paterson et al., 1995). In agriculture, the rhizome trait is of significance due to both its positive and negative effects. On the one hand, strong rhizomes largely contribute to the competitiveness and invasiveness of weeds (Jang et al., 2006). On the other hand, rhizomes are also dispersal in many forage crops and may be utilized for developing perennial grain crops (Hu et al., 2011). Notably, it has been speculated that genes controlling rhizome development could be used to prolong the nutritional life cycle of certain plant species, potentially converting major annual grain crops into perennial ones (Paterson et al., 1995; Hu et al., 2003; Cox et al., 2006; Glover et al., 2010). As such, understanding the genetic mechanisms underlying rhizome development is not only instrumental in devising control or productivity-enhancing strategies for rhizomatous plants, but also beneficial to sustainable food production and ecosystem maintenance given the multiple ecological threats posed by our current annual crop dominated agricultural system (Hu et al., 2003; Cox et al., 2006; Glover et al., 2010).

Previous efforts to dissect the genetic basis of the rhizome trait were largely hindered by the sparsity of genomic resources. Recently, *Oryza longistaminata* is emerging as a useful model system for exploring rhizome development (Hu et al., 2003; Li et al., 2022). Originated from Africa, *O. longistaminata* is the only rhizomatous wild *Oryza* species that has the same AA genome type as the cultivated rice species *O. sativa* (Morishima, 1967; Tao and Sripichitt, 2000; Hu et al., 2003; Sacks et al., 2003; Ghesquiere, 2008). Hence, it can be crossed with well-studied rice cultivars for which high-quality genomic information is available, a strategy that is widely used for mapping genes or quantitative trait loci (QTLs) for agronomic traits. It is noteworthy that through such strategy, known as wide hybridization, a number of perennial rice lines have been successfully developed and are currently on trial in many Chinese provinces, representing an environmentally and economically sound rice production system (Zhang et al., 2017, 2019; Huang et al., 2018; Zhang et al., 2021).

An early study (Meakawa et al., 1998) that took advantage of the hybridization between *O. sativa* and *O. longistaminata* has suggested that the rhizomatous growth habit of *O. longistaminata* was segregated as a single dominant trait. The single dominant allele responsible for the rhizome trait was termed *Rhz*, and it was loosely linked to the *liguleless* (*lg*) locus on chromosome 4 with a recombination value of 37 ± 3.6% (Meakawa et al., 1998). However, the rhizomatous phenotypes displayed pronounced variations in the F_2_ population, indicating the presence of other modifying genes. Based on a complete simple sequence-repeat map, Hu et al. (2003) identified two dominant-complementary loci, termed *Rhz2* and *Rhz3*, that predominantly control rhizomatousness in *O. longistaminata*, and the authors also revealed many QTLs affecting rhizome abundance (Hu et al., 2003). Recently, through entire population genotyping mapping and selective genotyping mapping using three F_2_ populations, over 10 major- or minor-effect rhizome-regulating QTLs were identified; however, none of these QTLs could be able to function alone, indicating that interactions among multiple QTLs are required for proper rhizome development (Fan et al., 2020). Nevertheless, to our knowledge, no reports have detailed how the rhizome-related QTLs interact with each other.

Understanding the relationships between complex genotypes and their underlying phenotypes is still one of the main challenges in modern genetics (Mackay et al., 2009). To better characterize the genetic networks underlying complex traits, a theoretical framework founded upon knowledge of signal transduction pathways has been proposed (Zhang et al., 2011). In this framework, the principle of hierarchy is defined as one-way functional dependency of downstream genes on upstream regulators, and functional genetic units (FGUs) refer to a group of functionally dependent genes acting at each level of a signaling pathway (Zhang et al., 2011). This framework was later employed to explore the pleiotropic effects of *SD1*, whose mutant alleles greatly contribute to the Green Revolution, and three genetic systems (*SD1*-mediated, -repressed, and -independent) were revealed, comprising 43, 38, and 64 FGUs, respectively, and jointly controlling growth, development and productivity of rice (Zhang et al., 2013). The functionality of this framework was further demonstrated in an investigation of the genetic basis of submergence tolerance (Wang et al., 2015). The putative networks consisted of 296 loci that were grouped into 167 FGUs, and the directional links between and among the nodes (the detected loci) suggested that submergence tolerance in rice is genetically controlled by a number of positively regulated signaling pathways (Wang et al., 2015).

Here, to gain insights into the genetic basis of rhizomatousness in *O. longistaminata*, a large F_2_ population containing 818 individuals was used to map loci that control various rhizome-related traits. These identified loci were further interwoven into putative networks to elucidate their interconnections. Our results highlight the intricate genetic regulation on rhizome development and may provide information for pinpointing key rhizome-related genes that may be utilized in future perennial rice breeding programs.

## Materials and Methods

### Plant Materials

The mapping population was prepared from a cross between an unnamed *O. longistaminata* accession featuring long and strong rhizomes and the *O. sativa* cultivar RD23 (an indica cultivar from Thailand) (Tao and Sripichitt, 2000). The F_1_ plant was grown at the Perennial Rice Research Station of Yunnan University located in Jinghong, Yunnan Province, China, a typically double rice cropping region with a tropical monsoon climate (20°57′ E, 100°45′ N, at an altitude of 555 m). A large number of F_2_ seeds were obtained by bagged self-pollination and vegetative propagation using tillers. All F_2_ plants were grown at the same region in Jinghong during the first cropping season of 2016, and the distance between F_2_ plants was 50 cm. At the time of flowering, all plants were dug up, and the underground parts were washed free of soil for phenotypic evaluation.

Recombinant inbred lines were developed in our laboratory using the single seed descent technique. Since 2007, one single individual plant from the previous generation was randomly selected for selfing. The seeds were harvested and planted as a new generation. These steps were repeated, and finally, a RIL population consisting of 133 lines was obtained. This RIL population was employed to preliminarily validate the key loci identified in this study.

### Phenotypic Evaluation

**FIGURE 1:**
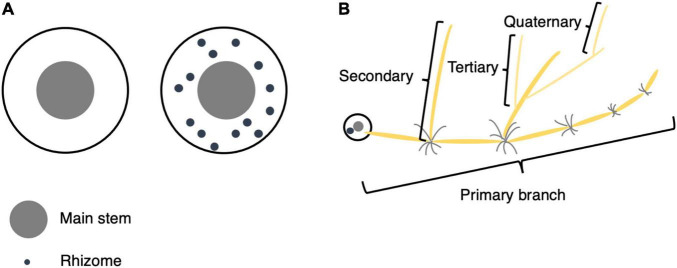
Phenotypic evaluation of rhizomes. **(A)** Top view of plants with and without rhizomes. If a plant’s underground part contained only the main stem and tillers, its Rhz was recorded as 0 (without rhizomes), and if there were obvious internodes in the underground part, its Rhz was recorded as 1 (with rhizomes). **(B)** Schematic illustration for primary, secondary, tertiary, and quaternary branches.

Each plant was first evaluated for the presence or absence of rhizomes (hereafter referred to as Rhz). For individuals with rhizomes, rhizome-related quantitative traits were measured, including rhizome number (RN), cumulative length of primary branches (RLsum), average length of primary branches (RLmean), maximum length of primary branches (RLmax), number of secondary branches (RB2), number of tertiary branches (RB3), and number of quaternary branches (RB4) per plant (Figure 1).

### Genotyping-by-Sequencing Library Construction and Single Nucleotide Polymorphism Detection

DNA was extracted using the cetyltrimethylammonium bromide method (Doyle, 1987). A genotyping-by-sequencing library was prepared following the protocol developed by Clark et al. (2014). Briefly, DNA samples were first digested by the restriction enzymes *Pst*I and *Msp*I, and then sheared to 200–400 bp for restriction site-associated DNA library construction. Sequencing was performed on an Illumina HiSeq 2000 machine and 150-base paired-end reads were obtained.

Raw read files were decoded for each sample by using “process_radtags” in Stacks v2.3 according to their barcode files. Subsequently, the raw reads for each sample were aligned to the *O. sativa* genome (IRGSP V1.0 *O. sativa* Nipponbare, Kawahara et al., 2013) by using the Burrows-Wheeler Alignment software (parameter: mem −t 4 −M −R). The alignment results were formatted and converted into input files using SAMtools. The programs “pstacks,” “cstacks,” and “genotypes” in Stacks were used to perform SNP calling and obtain the genotypic data based on SNPs. The raw SNPs were filtered by an R script. First, SNPs that were not present in over 20% of individuals in the population were filtered, and the second round of filtering was performed according to segregation ratios and parental genotypes: for each SNP, it should be homozygous in both RD23 and *O. longistaminata* genomes, and be able to differentiate *O. longistaminata* and RD23. Finally, 1665 high-quality SNPs were obtained for genetic map construction.

### Qualitative Trait Locus Identification and Quantitative Trait Loci Analysis

Chi-square tests were performed to detect qualitative trait loci associated with Rhz, with the significance level setting to 5%. On one chromosome, the locus with the maximum chi-square value was regarded as a hypothetical major-effect locus. To find all independent loci on the same chromosome, correlation analyses between the hypothetical major-effect locus and other loci were carried out. All loci that were significantly associated with the hypothetical main locus (*P* < 0.05) were considered as dependent loci and removed, and the remaining loci were identified as independent major-effect loci.

The linkage map was constructed by using Joinmap 4.0 (Van Ooijen, 2006). Inclusive composite interval mapping was performed for QTL analysis using QTL IciMapping 4.2 (Lei et al., 2015). The LOD score threshold for QTL identification was set to 2.50. QTLs were named as described by McCouch et al. (1997). Note that the corresponding QTLs for RLmean and RLmax were named as *qRLM* and *qRLMAX*, respectively.

### Network Construction

Using the molecular-quantitative genetic model developed by Zhang et al. (2011), a genetic network underlying each measured trait was constructed according to genetic expectations of the identified loci. Briefly, FGUs were identified by performing multiple comparisons of the corresponding phenotypic data of one genotype represented by two loci. When there was a significant phenotypic difference (*P* < 0.05) between one genotype and the other genotypes, and the interaction effect between the two loci was equivalent to the main effect, these two loci were grouped into an FGU. According to whether one locus was significant responsible for the phenotypical difference or not, the locus and the other locus in a locus pair were regarded as hierarchy QTLs (directional links) or epistatic QTLs/loci (E-QTLs/loci, non-directional links), respectively. The integrated network of all identified loci was assembled based on loci that were found to affect more than one rhizome-related trait.

### Verification of Key Quantitative Trait Loci

Locus sequences were obtained from the National Centre for Biotechnology Information and our unpublished *O. longistaminata* genome data (data not shown). Sequence-tagged site (STS) markers were designed by using Primer 5.0. The primer sequences were *qRHZ-3-1*-F (5′-CTACCAGGTTCGTT GATGTC-3′) and *qRHZ-3-1*-R (5′-CGAGGTACATCGTCTT GGA-3′) for *qRHZ-3-1*, *qRHZ-4*-F (5′-CAGACGGATTGAATC GATACCA-3′), and *qRHZ-4*-R (5′-CCATTTTCCCTGTTC ATCCATC-3′) for *qRHZ-4*, *qRHI-2*-F (5′-ATAAAATGGTATG GTGTAATGG-3′) and *qRHI-2*-R (5′-TGTTTCGCATTGCA TCTG-3′) for *qRHI-2*, *qRHI-5*-F (5′-TTAGCTCTCACAAA TGAATATC-3′), and *qRHI-5*-R (5′-TATCCAACCCTTCAA ACG-3′) for *qRHI-5*. PCR was performed in a 20 μL reaction containing 1 μL genomic DNA extracted from one RIL individual, 0.5 μL Primer-F/R, 2 μL 10 × Taq Buffer, 1.6 μL dNTP, 0.5 μL Taq polymerase, and 13.9 μL ddH_2_O. Amplification was programmed for 3 min at 94°C for initial denaturation and 30–35 cycles consisting of 30 s at 94°C, 30 s at 56–60°C, 20 s at 72°C, followed by a final 5 min at 72°C. Amplified DNA fragments were detected using 8% polyacrylamide gel electrophoresis.

## Results

### Phenotypes of the F_2_ Population

**FIGURE 2:**
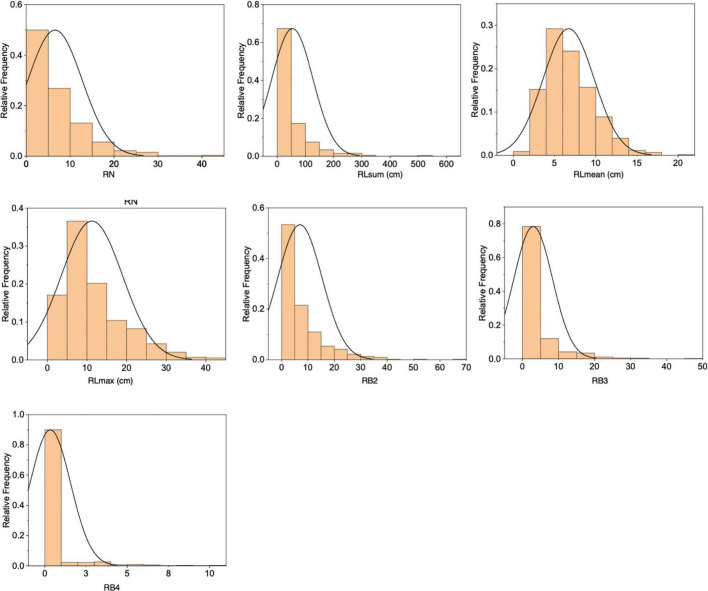
Distributions of seven rhizome-related traits in the F_2_ population.

The F_2_ population derived from a cross between RD23 and *O. longistaminata* contained 818 individuals, of which 585 individuals had rhizomes. Significant variations were observed in the seven measured rhizome-related traits (RN, RLsum, RLmean, RLmax, RB2, RB3, and RB4), all exhibiting a skewed distribution (Figure 2 and Supplementary Table 1). The correlation analysis of seven rhizome-related quantitative traits revealed that there were significant correlations between these traits, with the correlation coefficients of RN and RLsum, and RLmax and RLmean reached 0.934 and 0.873, respectively (Supplementary Table 2).

### Construction of Linkage Map of the F_2_ Population

**FIGURE 3:**
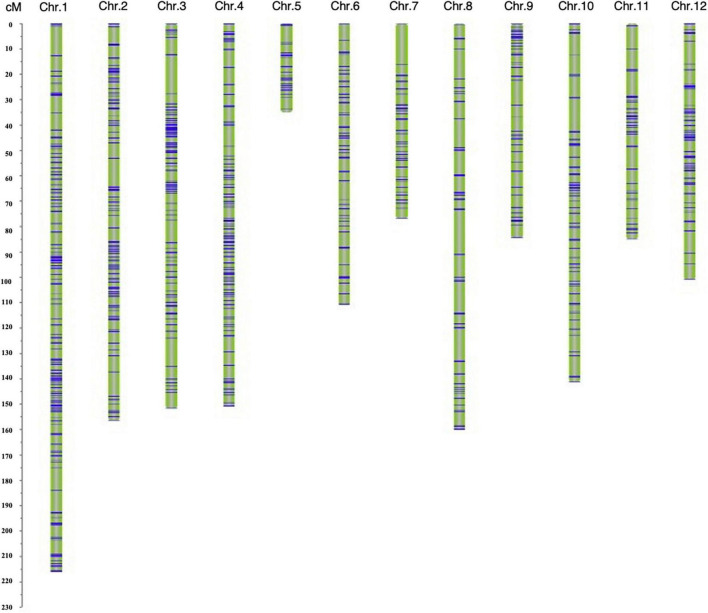
The genetic linkage map of the F_2_ population.

A genetic map was constructed based on 1487 SNP markers spanning 1469.27 cM and covering all 12 chromosomes. The length of a single chromosome was between 35.04 and 216.00 cM, and the average distance between makers was 0.99 cM (Supplementary Table 3). Among the 12 chromosomes, chromosome 1 harbored the largest number (267) of markers, and the average distance between markers was 0.81 cM, whereas the number of markers on chromosome 5 was the lowest (38 markers), with the average distance being 0.92 cM (Figure 3 and Supplementary Table 3).

### Qualitative Trait Loci for Rhz and Quantitative Trait Loci Affecting Rhizome Development

In all, 13 regions located in 12 chromosomes were identified as qualitative trait loci that were significantly associated with Rhz (Table 1 and Supplementary Table 4). Among these loci, chromosome 4-located *qRHZ-4* showed the most significant segregation distortion (χ^2^ = 119.16), and it was therefore hypothesized as one major Rhz locus. On this premise, the effects of the remaining 12 Rhz loci were further analyzed after the interference of *qRHZ-4* was removed. The results showed that, except for *qRHZ-2*, no other loci were significantly associated with Rhz. Hence, *qRHZ-2* could be regarded as another major hypothetical Rhz locus (Supplementary Table 5).

**TABLE 1:** Major-effect loci for Rhz.

Locus	Chr	Dis (cM)	Marker	Physical location	χ^2^	*P*
*qRHZ-1*	1	183.96	mk939	C0315063831	18.04	0.003
*qRHZ-2*	2	115.73	mk522	C026188261	33.69	0.000
*qRHZ-3-1*	3	38.19	mk876	C034764149	46.06	0.000
*qRHZ-3-2*	3	121.15	mk1010	C0335005202	14.75	0.012
*qRHZ-4*	4	116.23	mk1097	C0419314643	119.16	0.000
*qRHZ-5*	5	8.21	mk981	C0326657391	17.82	0.003
*qRHZ-6*	6	110.44	mk1347	C0617172779	14.33	0.014
*qRHZ-7*	7	27.76	mk1468	C0726307967	29.46	0.000
*qRHZ-8*	8	143.42	mk1536	C0824312935	15.22	0.009
*qRHZ-9*	9	1.44	mk468	C1120950915	13.12	0.022
*qRHZ-10*	10	138.8	mk115	C1023085906	33.85	0.000
*qRHZ-11*	11	42.79	mk420	C1110822965	24.04	0.000
*qRHZ-12*	12	60.94	mk793	C125003428	30.69	0.000

*Chr, chromosome; Dis, distance.*

**FIGURE 4:**
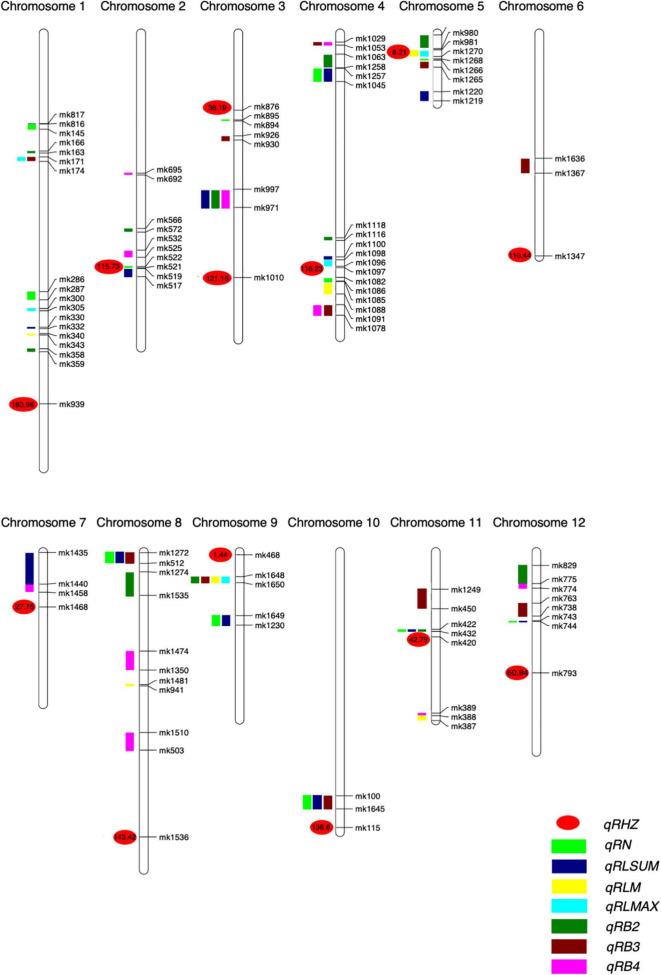
Positions of QTLs on the genetic linkage map of the F_2_ population. Numbers are genetic distances.

For the rhizome abundance traits measured in this study, a total of 51 QTLs were identified, while their effects on the phenotype varied greatly (Table 2 and Figure 4). For example, among the 12 QTLs that were found to affect RN, the highest phenotypic variation explained (PVE) was 10.45, whereas the lowest PVE was 1.38. Interestingly, we found that 11 QTLs may control at least two traits, and they were re-termed *qRHI*. Most of these QTLs appeared to affect multiple highly correlated traits, such as *qRHI-1* for RLmax and RB3; *qRHI-3* for RLsum, RB2, and RB4; *qRHI-4-1* and *qRHI-4-3* for RB3 and RB4; and *qRHI-4-2* for RLsum and RN. It is worth noting that three regions identified to determine Rhz were also found to be located in QTL intervals affecting other rhizome-related traits, such as *qRHI-2* for Rhz and RN, and *qRHI-5* for Rhz, RLmean and RLmax.

**TABLE 2:** Loci affecting rhizome-related traits in the F_2_ population.

QTL	Trait	Chr	Marker interval	LOD/χ^2^	PVE (%)	Add	Dom
*qRLSUM-1-1*	RLsum	1	mk817-mk816	8.29	3.94	22.87	0.45
*qRN-1-1*	RN	1	mk816-mk145	8.27	4.61	2.16	–0.05
*qRB2-1-1*	RB2	1	mk166-mk163	6.72	3.72	2.46	0.81
*qRHI-1*	RLmax	1	mk171-mk174	3.81	2.1	1.83	0.17
	RB3			5.68	3.57	1.66	0.06
*qRN-1-2*	RN	1	mk286-mk287	4.12	2.21	0.25	1.78
*qRLMAX-1-2*	RLmax	1	mk300-mk305	2.71	1.53	1.43	0.29
*qRLSUM-1-2*	RLsum	1	mk330-mk332	4.15	2.04	5.1	19.54
*qRLM-1*	RLmean	1	mk340-mk343	4.13	2.46	0.24	0.88
*qRB2-1-2*	RB2	1	mk358-mk359	2.8	1.54	–0.47	2.09
*qRHZ-1*	Rhz	1	mk939	18.04			
*qRB4-2*	RB4	2	mk695-mk692	34.27	42.62	3.01	–2.98
*qRB2-2*	RB2	2	mk566-mk572	4.79	7.95	14.31	–13.8
*qRB3-2*	RB3	2	mk532-mk525	23.05	27.32	9.26	–9.92
*qRHI-2*	Rhz	2	mk522-mk521	33.69			
	RN			15.55	8.45	3.6	–0.27
*qRLSUM-2*	RLsum	2	mk519-mk517	117.21	89.81	–143.39	17.44
*qRHZ-3-1*	Rhz	3	mk876	46.06			
*qRN-3*	RN	3	mk895-mk894	2.92	1.38	–2.1	–3.26
*qRB3-3*	RB3	3	mk926-mk930	4.39	3.1	–6.89	–7.42
*qRHI-3*	RLsum	3	mk997-mk971	2.56	7.88	–82.69	–89.46
	RB2			2.79	11.23	–8.1	–8.63
	RB4			53.94	47.99	–2.77	–2.76
*qRHZ-3-2*	Rhz	3	mk1010	14.75			
*qRHI-4-1*	RB3	4	mk1029-mk1053	15.48	22.31	9.11	–9.83
	RB4			58.04	47.95	3.26	–3.28
*qRB2-4-1*	RB2	4	mk1063-mk1258	6.04	11.26	11.44	–12.39
*qRHI-4-2*	RN	4	mk1257-mk1045	8.98	8.1	8.46	–8.73
	RLsum			19.76	13.76	166.4	–165.43
*qRB2-4-2*	RB2	4	mk1118-mk1116	11.67	6.66	3.13	0.49
*qRLSUM-4-2*	RLsum	4	mk1100-mk1098	8.87	4.38	22.56	–5.36
*qRLMAX-4*	RLmax	4	mk1098-mk1096	20.28	12.42	4	–0.72
*qRHZ-4*	Rhz	4	mk1097	119.16			
*qRN-4-2*	RN	4	mk1082-mk1086	6.3	3.12	1.56	–0.64
*qRLM-4*	RLmean	4	mk1085-mk1088	15.76	10.39	1.57	–0.23
*qRHI-4-3*	RB4	4	mk1091-mk1078	61.91	52.25	2.75	–2.9
	RB3		mk1091-mk1078	19.31	23.3	9.54	–10.08
*qRB2-5*	RB2	5	mk460-mk980	10.7	5.88	1.31	2.87
*qRHI-5*	Rhz	5	mk981-mk1270	17.82			
	RLmean			8.13	5.83	–0.12	1.63
	RLmax			6.84	4.19	0.99	2.22
*qRN-5*	RN	5	mk1268-mk1266	13.59	6.87	1.46	1.83
*qRB3-5*	RB3	5	mk1266-mk1265	7.08	13.66	7.23	–7.27
*qRLSUM-5*	RLsum	5	mk1220-mk1219	17.53	15.96	124.29	–129.87
*qRB3-6*	RB3	6	mk1636-mk1367	8.74	27.59	10.02	–10.1
*qRHZ-6*	Rhz	6	mk1347	14.33			
*qRLSUM-7*	RLsum	7	mk1435-mk1440	7.79	11.13	–104.8	–96.25
*qRB4-7*	RB4	7	mk1440-mk1458	26.37	27.89	–3.29	–3.07
*qRHZ-7*	Rhz	7	mk1468	29.46			
*qRHI-8*	RN	8	mk1272-mk512	7.34	8.27	8.92	–8.66
	RLsum			22.93	18.09	137.2	–137.76
	RB3			21.14	26.44	9.39	–9.37
*qRB2-8*	RB2	8	mk1274-mk1535	5.24	14.15	9.14	–9.65
*qRB4-8-1*	RB4	8	mk1474-mk1350	11.98	28.96	2.48	–2.65
*qRLM-8*	RLmean	8	mk1481-mk941	3.13	1.92	–1	0.22
*qRB4-8-2*	RB4	8	mk1510-mk503	88.25	61.16	2.76	–2.79
*qRHZ-8*	Rhz	8	mk1536	15.22			
*qRHZ-9*	Rhz	9	mk468	13.12			
*qRHI-9-1*	RB2	9	mk1648-mk1650	5.07	7.77	8.84	–7.5
	RB3			9.47	20.66	11.62	–11.18
	RLmean			4.38	2.78	1.66	–0.87
	RLmax			5.12	2.93	3.34	–1
*qRHI-9-2*	RN	9	mk1649-mk1230	6.31	10.45	6.62	–6.89
	RLsum			22.03	16.98	127.18	–128.99
*qRHI-10*	RN	10	mk100-mk1645	5.86	8.68	4.45	–3.64
	RLsum			13.94	16.33	77.3	–68.09
	RB3			4.2	21.25	6.43	–5.68
*qRHZ-10*	Rhz	10	mk115	33.85			
*qRB3-11*	RB3	11	mk1249-mk450	10.3	21.34	10.33	–10.61
*qRHI-11*	RN	11	mk422-mk432	9.94	5.12	–1.92	–0.5
	RLsum			6.94	3.33	–18.35	–4.95
	RB2			6.91	3.76	–2.27	–0.5
*qRHZ-11*	Rhz	11	mk420	24.04			
*qRB4-11*	RB4	11	mk389-mk388	3.42	2.8	0.24	–0.3
*qRLM-11*	RLmean	11	mk388-mk387	2.69	1.75	0.57	–0.22
*qRB2-12*	RB2	12	mk829-mk775	5.53	11.96	–8.73	–6.84
*qRB4-12*	RB4	12	mk775-mk774	8.39	12.21	–2.73	–2.78
*qRB3-12*	RB3	12	mk763-mk738	2.56	6.49	–6.53	–6.15
*qRHI-12*	RN	12	mk743-mk744	5.37	3.82	–14.36	–13.82
	RLsum			5.53	3.73	–169.15	–163.57
*qRHZ-12*	Rhz	12	mk793	30.69			

*Chr, chromosome; PVE, phenotypic variation explained; Add and Dom, QTL additive and dominance effects, respectively.*

### *qRHZ-3-1*, *qRHZ-4*, *qRHI-2*, and *qRHI-5* Are Key Quantitative Trait Loci in the Genetic Network Underlying Rhizome Development

**FIGURE 5:**
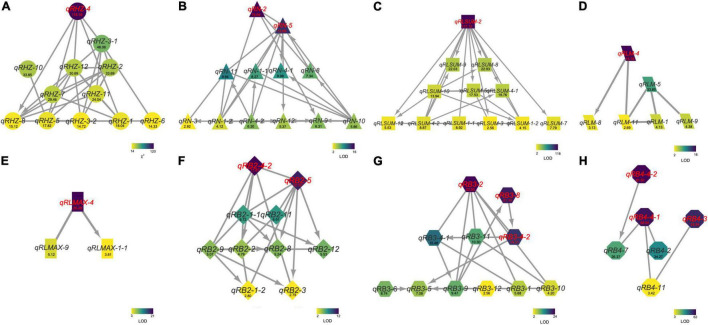
The genetic networks for Rhz **(A)**, RN **(B)**, RLsum **(C)**, RLmean **(D)**, RLmax **(E)**, RB2 **(F)**, RB3 **(G)**, and RB4 **(H)**. A line between two nodes indicates that these two nodes are a pair of E-loci **(A)** or E-QTLs **(B–H)**. An arrow between two nodes represents a hierarchical relationship between this node pair, with the node at the arrowhead being regulated by the other node of the pair. Numbers are Chi square values **(A)** or LOD values **(B–H)**.

To further understand the relationships between and among the identified loci, we constructed a genetic network for each trait based on FGUs (Figure 5 and Supplementary Table 6). A total of 12 FGUs affecting Rhz were detected, consisting of 2 pairs of hierarchical loci and 29 pairs of E-loci (Figure 5A and Supplementary Table 6). This network displayed a clear hierarchical structure with *qRHZ-4* as the highest-level node. Another similar highly hierarchical network was the RLsum network, with *qRLsum* as the top node and each layer containing more nodes along with the hierarchy (Figure 5C). By contrast, the networks for RB and RB4 appeared to be non-hierarchical, with no obvious loci located on the upstream of other loci (Figures 5F,H). The network for RLmax was the network of the simplest form (containing only three nodes), in which *qRLmax-4* was hierarchically connected with other two nodes (Figure 5E).

**FIGURE 6:**
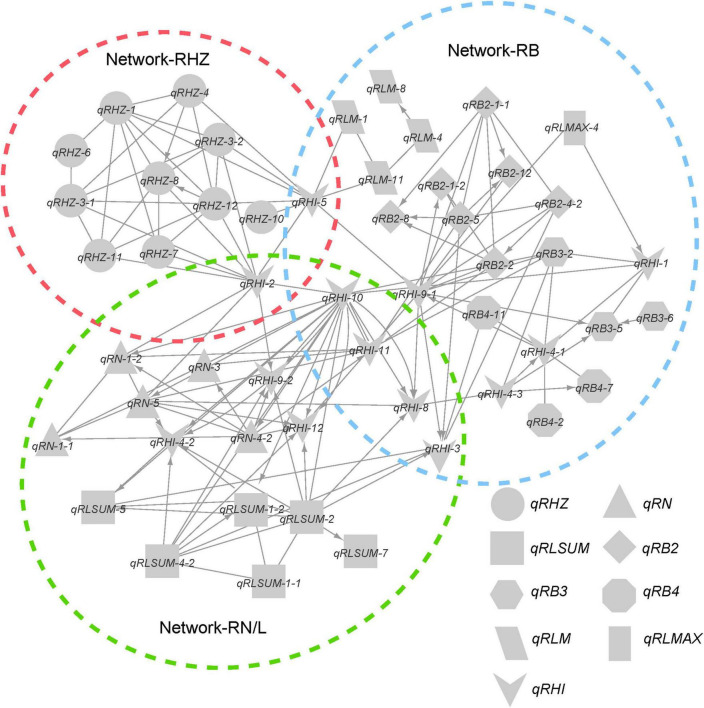
Genetic network underlying rhizome development.

Considering the possible interactions among loci for different traits, we further integrated the individual networks for single traits into a comprehensive network (Figure 6). In this network, a total of 58 FGUs were included, containing 62 pairs of hierarchy loci/QTLs and 104 pairs of E-loci/QTLs. Based on network properties, we extracted three sub-networks and termed them as network-RHZ, network-RB, and network-RN/L, which corresponded to Rhz, rhizome branching traits, and rhizome number/length, respectively. Given that network-RHZ should be the prerequisite for other two networks, its nodes with the most marked effects, *qRHZ-3-1* and *qRHZ-4*, and its connector nodes with the other two networks, *qRHI-2* and *qRHI-5* were classified as key loci in the network (Figure 6).

### Preliminary Verification of Key Loci in the Networks

**FIGURE 7:**
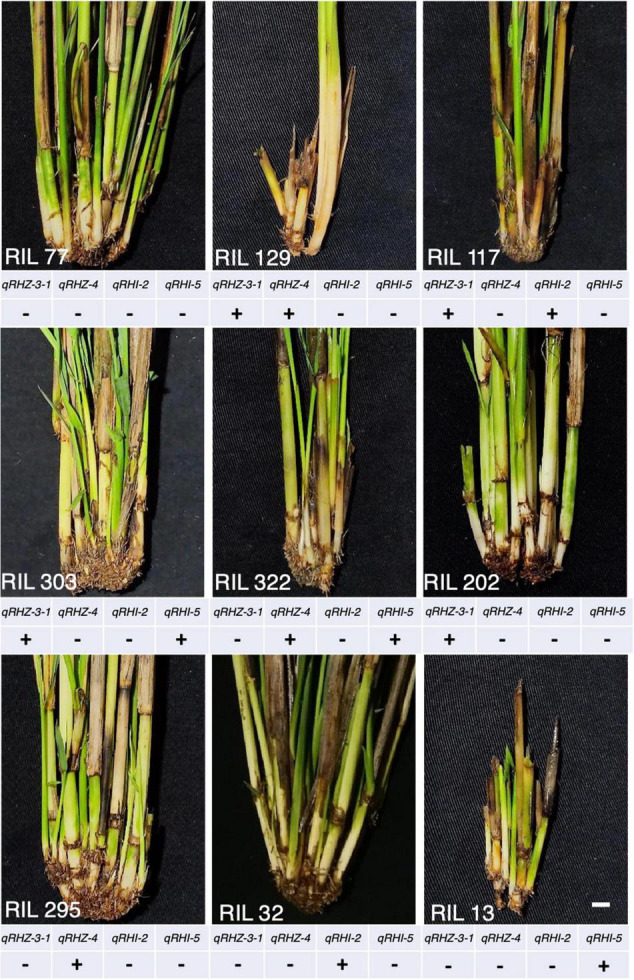
Phenotypes of representative rhizome-free RILs. “+” and “–” represent *O. longistaminata* and RD23 homozygous alleles at the locus, respectively.

To preliminarily verify the biological function of the four key loci we identified, we detected the genotypes of a rhizome-free RIL population containing 133 individuals by using the STS markers of these loci, and observed the corresponding phenotypes. For each individual locus, most individuals of the RIL population were RD23 homozygous, but a few *O. longistaminata* homozygous individuals were also detected (Supplementary Table 7). There were five individuals harboring two pairs of *O. longistaminata* homozygous alleles, namely, RIL 129, 117, 303, 304, and 322 (Figure 7; as RIL 303 and 304 are of the same genotype, only the phenotype of RIL 303 is shown). The observation that all RILs with homozygous *O. longistaminata* alleles at single locus were rhizome-free confirmed that no single locus can ensure the presence of rhizomes. Moreover, none of these rhizome-free individuals were detected to harbor more than two of these key loci with *O. longistaminata* alleles, suggesting that three or more loci with *O. longistaminata* alleles are required for the presence of rhizomes. Given that we failed to detect any genotype with homozygous *O. longistaminata* alleles at both *qRHI-2* and *qRHI-5*, two multi-trait controlling loci that appeared to be critical in our network analyses, we hypothesize that these two loci are likely both needed for proper rhizome growth.

## Discussion

In the present study, we substantially expanded the genetic repertoire for rhizome development. A recent manuscript (Fan et al., 2020) identified over 10 loci related to rhizome growth, including five major-effect loci—*qRED1.2*, *qRED3.1*, *qRED3.3*, *qRED4.1*, and *qRED4.2*, some of which are partially overlapped with two previously mapped loci determining the presence of rhizome, *Rhz2* and *Rhz3* (Hu et al., 2003). Here, we identified 13 Rhz-regulating loci and as many as 51 QTLs controlling rhizome abundance. Not surprisingly, some of them are overlapped with those previously reported QTLs. Comparing with Fan et al. (2020), eight of our identified loci (*qRN-1-2*, *qRLSUM-2*, *qRHZ-3-1*, *qRHZ-3-1*, *qRHI-3*, *qRB2-4-2*, *qRHI-11*, and *qRHZ-11*) are overlapped with their QTL intervals. Among these, two Rhz-regulating loci, *qRHZ-3-1* and *qRHZ-11*, are located in the intervals of *qRED3.1* and *qRED-11*, respectively, highlighting the potential prerequisite role of these loci, and *qRHI-3* and *qRHI-11*, two intervals controlling multiple rhizome traits, were found to coincide with *qRED3.3* and *qRED11* (Fan et al., 2020). Meanwhile, many rhizome abundance QTLs we identified are closely linked with their major-effect loci, such as *qRB2-2* and *qRED2.2*, *qRN-3* and *qRED3.1*, and *qRLSUM-4-2* and *qRED4.1* (Fan et al., 2020). In general, these two studies are mutually confirmative; however, it is important to note that, instead of using the average rhizome extension distance (measured in soil surface, Fan et al., 2020), we treated the presence of rhizomes as a qualitative trait and carefully evaluated specific rhizome-related traits by digging the plants up. This may explain why our results do not completely include the previously identified QTLs, and such comprehensive phenotypic evaluation also evidently gave rise a larger number of identified QTLs in our study. Furthermore, the results presented here are also largely consistent with our previous study (Hu et al., 2003). A total of six pairs of loci are overlapped, and many of these loci pairs were identified to control the same or similar trait, such as *qRN-1-2* and *QRl1*, and *qRBD3-2* and *QRbd2*. The disparity between our current and previous results may stem from the distinct populations. Moreover, compared with previously used simple-sequence repeat (Hu et al., 2003) and insertion/deletion markers (Fan et al., 2020), the SNP markers used in this study have relatively wider coverage and higher density in the whole genome; thus, more QTLs have emerged.

Multiple genetic networks were established on the basis of classical QTL mapping (Zhang et al., 2011; Figures 5, 6). We observed a clear hierarchical structure in some networks for individual traits (Figure 5). This indicates that some loci, such as *qRHZ-4* in the Rhz network (Figure 5A) and *qRLsum-2* in the RLsum network (Figure 5C), may play a more significant regulatory role than the other loci in the same network since upstream loci are likely prerequisite for downstream loci to execute their functions. Given that the other traits measured in this study all depend on the presence of rhizomes, the two top-located nodes in the RHZ network are presumably of primary importance for proper rhizome development. Furthermore, in the comprehensive network containing all the identified loci, *qRHI-2* and *qRHI-5* were the hub loci connecting network-RHZ with network-RB and network-RN/L. In network analyses, hub nodes are generally considered to be critical (Zhang et al., 2011). In our case, considering that no other networks would exist if network-RHZ collapses, *qRHI-2* and *qRHI-5* are therefore considered as secondarily important loci for rhizome development. It is attempting to speculate that these four loci might be the minimal set of loci required for rhizome development, and this is worth of further investigation. Of note, the concept of FGU in this study is defined as a pair of mutual functional dependent genes, whose encoding proteins may not necessarily physically interact with each other, and FGUs in our networks were generated by comparing the genes’ contributions to the phenotype. Compared with RIL and doubled haploid populations, F_2_ populations usually have a larger number of distinct genotypes, and the efficacy of detecting genetic networks using F_2_ populations is weakened due to the possible errors occurred in multiple comparisons (Zhang et al., 2011). Such weaking effect would be amplified if there are a large number of loci controlling the trait (Zhang et al., 2011). Therefore, even though our preliminary functional verification suggested that the two hub loci, *qRHI-2* and *qRHI-5*, are likely both needed for the rhizome phenotype, we could not exclude the possibility that other loci are also required here. Further verification using rhizomatous accessions and functional characterization of these loci would be needed to unravel the complex genetic basis of rhizome development.

## Conclusion

In this study, we detected 62 loci that are putative regulators for rhizome development. By grouping these loci into FGUs, we provided evidence that rhizome growth is controlled by a multi-locus, multi-layered genetic network. Based upon the network structure and the interactions among the nodes, we predicted four loci, two major-effect loci for rhizome initiation and two hub loci connecting the individual genetic networks, as key loci. Functional verification using rhizome-free RILs confirmed that none of these key loci could solely initiate rhizome morphogenesis and suggested that *qRHI-2* and *qRHI-5* are likely both needed for rhizome initiation.

## Data Availability Statement

The original contributions presented in the study are included in the article/Supplementary Material, further inquiries can be directed to the corresponding authors.

## Author Contributions

FH and SZ designed the experiments. WL, SZ, LH, GH, and JZ performed the experiments. FH, ZL, and WL wrote the manuscript. WL, SZ, and ZL analyzed the data. All authors have read and approved the final manuscript.

## Conflict of Interest

The authors declare that the research was conducted in the absence of any commercial or financial relationships that could be construed as a potential conflict of interest.

## Publisher’s Note

All claims expressed in this article are solely those of the authors and do not necessarily represent those of their affiliated organizations, or those of the publisher, the editors and the reviewers. Any product that may be evaluated in this article, or claim that may be made by its manufacturer, is not guaranteed or endorsed by the publisher.
